# GLS2 shapes ferroptosis in hepatocellular carcinoma

**DOI:** 10.18632/oncotarget.28526

**Published:** 2023-10-19

**Authors:** Sawako Suzuki, Divya Venkatesh, Tomoaki Tanaka, Carol Prives

**Keywords:** ferroptosis, hepatocellular carcinoma, GLS2, p53, tumor suppression

More than a decade has passed since our group (1) as well as Hu et al., (2) identified glutaminase (GLS2) as a p53 target gene that promotes the tricarboxylic acid cycle (TCA) via α-ketoglutarate (αKG) and lowers oxidative stress via increasing glutathione (GSH) [1, 2]. Two years after this Dixon et al., [3] described a form of cell death they named ferroptosis which is caused by iron-mediated lipid peroxidation. Then, three years later, Gao et al., reported that GLS2 but not GLS1 is an inducer of ferroptosis in human cancer cells [4]. Ferroptosis had first been shown to be regulated by p53 via repression of SLC7A11 [5]. The circle was closed by a study from the Murphy group who reported that a cancer-related non- synonymous mutation in p53 (P47S) is correlated with to failure to either activate GLS2 expression or produce ferroptosis [6].

Our recent study (Suzuki et al.) [7] has validated the ability of GLS2 to promote ferroptosis in murine models. Questions also been posed as to why GLS2 but not GLS1 is required for ferroptosis given that they both have glutaminase core domains and can regulate glutaminolysis. Further, given that ferroptotic cell death occurs as a result of breakdown in cellular oxidative homeostasis, it had been difficult to reconcile this with a previously described a role for GLS2 as an antioxidant factor. Our work has now provided evidence that GLS2 is mainly localized in mitochondria and induces ferroptosis through α-ketoglutarate (αKG), and this occurs specifically under conditions where the levels of GSH or of glutathione peroxidase 4 (GPX4) are suppressed by ferroptosis inducers [7]. Given these findings, it is possible that the different intracellular localizations of mitochondrial GLS2 and cytosolic GLS1 may contribute to their different ferroptosis-promoting abilities.

**Figure 1:**
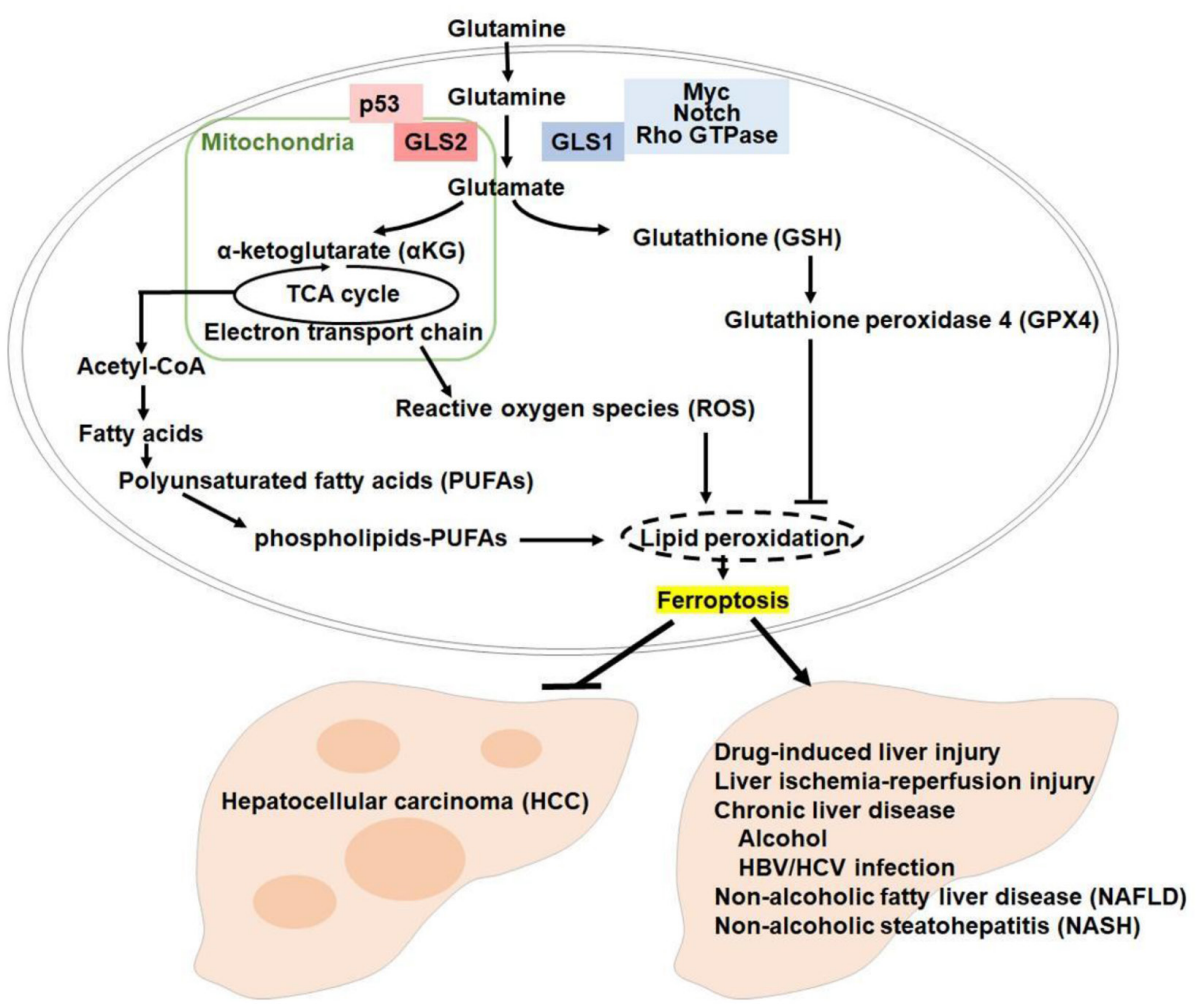
Ferroptosis regulation of GLS2 is a potential therapeutic strategy against liver diseases.

Our results also suggest the existence of a potential switch in the functional roles of GLS2 based on the needs of the cell [7]. We speculate that under normal unstressed conditions, GLS2 promotes the TCA cycle in the mitochondria via αKG and, at the same time, protects against accumulation of ROS by catalyzing increased levels of GSH. This balance would be loosened in some states that are prone to ferroptosis, including the downregulation of antioxidants or upregulation of ferroptosis execution factors such as iron, fatty acids, and ROS. Thus, in such situations of acute stress, the GLS2-αKG arc may switch to supplying fatty acids (polyunsaturated fatty acids and phospholipids) via the TCA cycle. Higher TCA cycle intermediates would also increase cellular free electrons through the more active electron transport chain and, given the presence of oxidizable lipids, this might result in the upregulation of lipid peroxidation that promotes ferroptosis (Figure 1).

Prior to our study it was somewhat controversial as to whether GLS2 is a *bona fide* tumor suppressor at an organismal level. Importantly, we found that loss of GLS2 *in vivo* is associated with the development of hepatocellular carcinoma (HCC) [7]. The absence of proper preventive or curative treatments for HCC has led to new first line therapeutic strategies including a tyrosine kinase inhibitor in 2009 [8], a molecular target agent that inhibits VEGFR in 2018 [9], and immune checkpoint inhibitor in 2020 [10]. Unfortunately, these treatments not been satisfactory due to their toxicities and rapid drug resistance and so new therapies that target different signaling pathways are urgently required. As the liver contains high concentrations of iron and is an important site of lipid metabolism, liver disease is a critical ferroptosis-associated disorder, [11]. In fact, ferroptosis is attracting attention as a therapeutic target for HCCs that are especially resistant to traditional treatments, although there are scant few molecules that can induce ferroptosis clinically other than two chemotherapeutics (sorafenib and sulfasalazine) that have been shown to possess ferroptotic abilities in addition to their primary mechanisms of action [12]. GLS2 is most abundant in the liver, and GLS2 downregulation is associated with HCC development. Therefore, we propose that the GLS2-αKG-ferroptosis axis might lay the foundation for a novel therapeutic approach for HCC [7].

**Figure 2:**
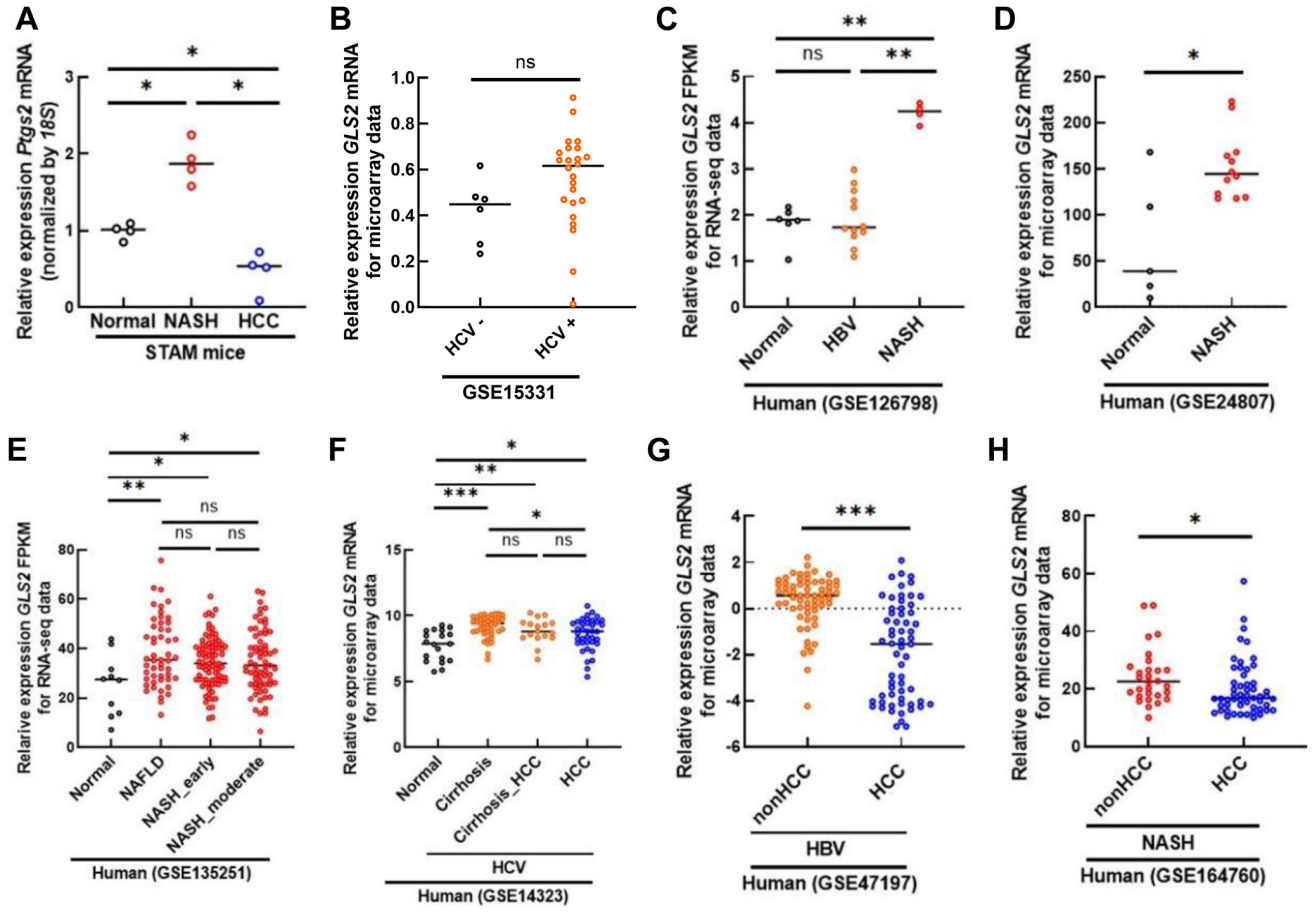
GLS2 expression in liver diseases. (**A**) Expression levels of *Gls2* (mRNA) in normal liver (*n* = 4), non-alcoholic steatohepatitis (NASH; *n* = 4) and HCCs of Stelic Animal Model (STAM; *n* = 4) mice were determined by the comparative threshold cycle method and then normalized to 18S expression. ^*^ *p* < 0.05. (**B**–**H**) Two RNAseq datasets (GSE126798 and GSE135251) and five microarray datasets (GSE15331, GSE24807 GSE14323, GSE47197 and GSE164760) were systematically extracted from the GEO database. The GLS2 gene expression in human liver samples from each dataset was quantified. GSE15331 in (B) is comprised of 6 hepatitis C virus negative (HCV−) and 24 negative (HCV−) liver samples. GSE126798 in (C) includes 6 healthy liver (normal), 12 HBV and 4 NASH. GSE24807 in (D) includes 5 normal and 12 NASH. GSE135251 in (E) includes 10 normal, 51 NAFLD, 87 NASH with mild fibrosis (NASH_early) and 68 NASH with moderate fibrosis (NASH_moderate). GSE14323 in (F) includes 19 normal, 41 HCV-associated cirrhosis, 17 HCV-associated cirrhosis with HCC and 38 HCC. GSE47197 in (G) is comprised of 63 HBV infected non tumoral (nonHCC) and 61 tumors (HCC). GSE164760 in (H) is comprised of 53 NASH-associated HCC (HCC) liver tissues and 29 adjacent non-tumor NASH (nonHCC). In statistics, the Mann-Whitney *U* Test was used to compare samples and *P* < 0.05 was considered statistically significant. ^***^ *p* < 0.001, ^**^ *p* < 0.01,^*^ *p* < 0.05. Abbreviation: ns: not significant.

On the other hand, it is important to consider the ramifications of our finding connecting that GLS2 with ferroptosis in the liver. Ferroptosis has also been proposed as key to the pathogenesis of several agents that cause liver damage, including drug-induced liver injury caused by acetaminophen, liver ischemia-reperfusion injury induced by shock or after surgery, chronic liver disease resulting from excess alcohol consumption or hepatitis B virus (HBV)/hepatitis C virus (HCV) infection, as well as non-alcoholic fatty liver disease (NAFLD) and non-alcoholic steatohepatitis (NASH) [11]. Since HCC can result from chronic HBV/HCV infection or NAFLD/NASH, we examined the GLS2 expression profiles in each stage of our STAM mouse model for NASH and HCC and found that Gls2 mRNA levels were actually decreased in the HCCs, but were markedly increased at the NASH stages (Figure 2A). In agreement with the results we obtained from mice, examining the Gene Expression Omnibus (GEO) database revealed that the mRNA levels of GLS2 are not significantly different in human hepatitis B/C-infected liver tissues when compared to healthy liver (Figure 2B and 2C), while being significantly higher in human NAFLD/NASH and liver cirrhosis (Figure 2C–2F). Although it is unclear why GLS2 expression is increased in NALD/NASH and liver cirrhosis, GLS2 is a p53 target, so the p53 status may alter in these stages. Note that significantly lower GLS2 gene expression was observed in HCC [7] regardless of the etiologies of background liver disease such as HBV/HCV infection or NASH (Figure 2F–2H). We also found hypermethylation of CpG islands in the Gls2 promoter that most likely results in the downregulation of Gls2 in HCC [7]. This might be a case of cancer cells hijacking a mechanism that may have been originally meant to protect against liver damage due to GLS2-induced ferroptosis.

While our findings of the GLS2-αKG-ferroptosis network might lay the foundation for a novel therapeutic approach for HCC, drug development efforts would require consideration of several potentially influencing factors. For example, some p53 mutations such as p53^3KR^(K117R+K161R+K162R) retain the ability to activate GLS2 and to function as a tumor suppressor [13], whereas another mutant form of p53(P47S) can downregulate GLS2 expression and is resistant to ferroptosis [6, 14]. Determining the type of tumors that harbor certain p53 mutations (or GLS2 loss-of-function mutations) may be important in choosing the right context for ferroptotic therapy. Additionally, given our above discussion about the dichotomous roles of ferroptosis in the liver, there probably exists a fine balance between curbing HCC progression and promoting liver damage when the GLS2-αKG-ferroptosis network is chemically enhanced. Since GLS2 is increased in human NAFLD/NASH and liver cirrhosis, further examination of whether GLS2 drives or protects other ferroptosis-associated liver diseases is essential when targeting ferroptosis for HCC treatment. If indeed GLS2 can promote chemically-induced ferroptosis irrespective of the tissue type, then the drug regimen will need to be tailored such that the liver tissues adjacent to HCC are protected. Taking these concerns into consideration, we hope that our findings will inform future decisions regarding treatment of liver disease.
